# Effects of Preoperative Glucocorticoid Use on Patients Undergoing Single-Level Lumbar Fusions: A Retrospective Propensity Score-Matched Registry Study

**DOI:** 10.7759/cureus.57197

**Published:** 2024-03-29

**Authors:** Mason T Stoltzfus, Kenny Nguyen, Zachary Freedman, David R Hallan, Jinpyo Hong, Elias Rizk

**Affiliations:** 1 Department of Neurosurgery, Penn State University College of Medicine, Milton S. Hershey Medical Center, Hershey, USA

**Keywords:** sepsis, thromboembolic event, surgical tracheostomy, nosocomial pneumonia, renal insufficiency, unplanned reoperation, surgical site infection(ssi), surgical complication, spinal fusion surgery, preoperative glucocorticoids

## Abstract

Objective

Spinal fusions are gaining popularity as a means of treating spinal deformity and instability from a range of pathologies. The prevalence of glucocorticoid use has also increased in recent decades, and their systemic effects are well-documented. Although commonly used in the preoperative period, the effects of steroids on outcomes among patients undergoing spinal fusions are inadequately described. This study compares the odds of developing complications among patients who underwent single-level lumbar fusions with and without preoperative glucocorticoid use in hopes of establishing more evidence-based parameters for guiding preoperative steroid use.

Methods

The TriNetX multi-institutional electronic health record database was used to perform a retrospective, propensity score-matched analysis of clinical outcomes of two cohorts of patients who underwent posterior or posterolateral single-level lumbar fusions with and without interbody fusion, those who used glucocorticoids for at least one week within a year of fusion and those who did not. The outcomes of interest were examined within 30 days of the operation and included death, reoperation, deep or superficial surgical site infection (SSI), pneumonia, reintubation, ventilator dependence, tracheostomy, acute kidney injury (AKI), renal insufficiency, pulmonary embolism (PE) or deep venous thrombosis (DVT), urinary tract infection (UTI), emergency department (ED) visit, sepsis, and myocardial infarction (MI).

Results

The odds of developing pneumonia within 30 days of spinal fusion in the cohort that used glucocorticoids within one year of operation compared to the cohort without glucocorticoid use was 0.67 (p≤0.001, 95% CI: 0.59-0.69). The odds of requiring a tracheostomy within 30 days of spinal fusion in the cohort that used glucocorticoids within one year of operation compared to the cohort without glucocorticoid use was 0.39 (p≤0.001, 95% CI: 0.26-0.60). The odds of reoperation, deep and superficial SSI, and ED visits within 30 days of operation were significantly higher for the same glucocorticoid-receiving cohort, with odds ratios of 1.4 (p=0.003, 95% CI: 1.11-1.65), 1.86 (p≤0.001, 95% CI: 1.31-2.63), 2.28 (p≤0.001, 95% CI: 1.57-3.31), and 1.25 (p≤0.001, 95% CI: 1.17-1.33), respectively. After propensity score-matching, there was no significant difference between the odds of death, DVT, PE, MI, UTI, AKI, sepsis, reintubation, and ventilator dependence between the two cohorts.

Conclusion

In support of much of the current literature regarding preoperative glucocorticoid use and rates of complications, patients who underwent a single-level lumbar fusion and have used glucocorticoids for at least a week within a year of operation experienced significantly higher odds of reoperation, deep and superficial SSI, and ED visits. However, these patients using glucocorticoids were also found to have lower odds of developing pneumonia, renal insufficiency, and tracheostomy requirement than those who did not use steroids within a year of surgery.

## Introduction

Over the past several decades, there has been an increase in the prevalence of spinal fusion procedures worldwide due to advances in equipment, techniques, surgical approaches, and the availability of new implant and graft materials [1]. These advances in spinal fusion surgery have expanded its indications to include spinal instability and deformity secondary to tuberculosis, scoliosis, traumatic spondylolisthesis, spinal stenosis, instability due to tumors, pseudoarthrosis, and congenital or degenerative disc disease, with lumbar degenerative disc disease the most common indication for spinal fusion [2]. Between 1998 and 2008, utilization of spinal fusion increased at a higher rate than other inpatient procedures, including laminectomy, hip replacement, knee arthroplasty, and percutaneous coronary angioplasty. Additionally, in-hospital mortality rates for spinal fusion have decreased despite increasing the average age of recipient patients [3].

Glucocorticoid medication use has dramatically increased since its discovery in the 1940s due to its well-demonstrated anti-inflammatory effects [4]. Data collected through National Health and Nutrition Surveys estimated the prevalence of glucocorticoid use in the US population to be 1.2%, or 2.5 million people, with a mean duration of use of 1605 days. Nearly 29% of those users undergo chronic therapy lasting greater than five years [5]. Despite the beneficial anti-inflammatory properties of corticosteroids, a significant correlation between chronic steroid use and surgical complications has been reported [6]. Complications associated with preoperative glucocorticoid use include perioperative diabetes, bleeding disorders, and sepsis, and have been demonstrated across a range of surgical specialties, including gastrointestinal surgery, neurosurgery, and orthopedic surgery [7-11]. In the specific area of lumbar spinal surgery, studies have demonstrated increased rates of reoperation, sepsis, urinary tract infections (UTIs), thromboembolic events, and more among patients who used glucocorticoids prior to surgery. However, there has been limited study of the effects of preoperative glucocorticoids on outcomes following elective spinal fusions, specifically [12,13]. A recent study by Mahmoodkhani et al. demonstrated improved long-term functional and neurological outcomes among spinal trauma patients undergoing instrumented fusions at the thoracolumbar junction who received perioperative steroids [14].

Considering these conflicting results, as well as gaps in the literature, more research must be dedicated to examining preoperative glucocorticoid use and its association with patient mortality and perioperative complications for a commonly performed procedure like elective spinal fusion surgery [12-14]. There is a paucity of studies looking at 30-day perioperative complications associated with chronic steroid use among patients prior to undergoing elective lumbar fusion. The primary objective of this study is to determine if chronic, preoperative glucocorticoid therapy provided any harmful or protective effects for patients undergoing single-level lumbar spinal fusion operations.

## Materials and methods

Study design

This retrospective cohort study utilized the TriNetX Research Network database, which encompasses 69 healthcare networks across the world, including over 106 million patients, to query for patients who underwent lumbar fusion operations using the International Classification of Diseases, 10th Revision (ICD-10) and current procedural terminology (CPT) codes systems. TriNetX is a multi-institutional, de-identified patient database derived from electronic medical records worldwide. No Institutional Review Board (IRB) approval or patient consent was needed due to the de-identified nature of the database. Previous literature confirmed the validity of this database, and its exact details are well-described [15-17].

The criteria of glucocorticoid use within one year of operation were selected because glucocorticoids have been demonstrated to exert downstream effects, causing an imbalance of endogenous glucocorticoid production by the hypothalamic pituitary adrenal axis, for up to a year [18].

Patient selection

The TriNetX database was queried for patients over 18 who underwent a single-level posterior or posterolateral lumbar fusion with or without interbody fusion using both allografts and autografts with or without the use of bone morphogenic proteins for the treatment of spinal instability or deformity. This patient selection process, using CPT codes 22612, 22630, and 22633, was chosen to remain comparable to previously published literature [19]. Patients were divided into two cohorts: those that were using glucocorticoids for at least a week, including betamethasone, cortisone, dexamethasone, hydrocortisone, methylprednisolone, prednisolone, and prednisone, within one year before the operation, and those that did not use glucocorticoids during this period. The resulting patients were then reviewed for preoperative covariates, including age, sex, demographics, BMI, osteopenia, osteoporosis, diabetes mellitus, nicotine dependence, hypertension, heart failure, chronic obstructive pulmonary disease, kidney failure, obesity, and underweight, as identified by their respective ICD codes. Both groups were then propensity score-matched to construct an artificial control cohort and a matched experimental cohort. This method accounts for covariates in an attempt to mimic true randomization as much as possible with a retrospective, observational study.

Statistical analysis

The primary statistical tool used in this study is an odds ratio, which measures the association between exposure and outcome. An odds ratio quantifies the odds of an outcome occurring following exposure to a variable, compared to the odds of that outcome occurring without exposure. In this study, preoperative glucocorticoid use was the exposure. Primary postoperative outcomes were measured for both cohorts within 30 days of operation and included death, reoperation, deep and superficial surgical site infection (SSI), pneumonia, reintubation, ventilator dependence, tracheostomy, acute kidney injury (AKI), renal insufficiency, pulmonary embolism (PE) or deep venous thrombosis (DVT), UTI, emergency department (ED) visit, sepsis, and myocardial infarction (MI). The outcome of reoperation within 30 days serves as a proxy catching indications for early reoperation, such as wound dehiscence and cerebrospinal fluid leakage. Importantly, this 30-day time limit excludes late indications for reoperation, such as adjacent segment disease or other degenerative sequela of instrumented lumbar fusions.

The data was analyzed through the TriNetX software using JAVA, R, and Python programming languages. Statistical significance was determined by an alpha level of 0.05.

## Results

Demographics

In total, 67741 patients were identified in the glucocorticoid group, while 26938 patients were identified in the non-glucocorticoid group. At the time of surgery, the average patient ages were 61 and 59 years in the glucocorticoid and non-glucocorticoid cohorts, respectively (p<0.001). After matching, 26903 patients with an average age of 59 years remained in both cohorts (p=0.56). 52% of the glucocorticoid cohort and approximately 51% of the non-glucocorticoid cohort were female and about 83% in the glucocorticoid cohort and 82% in the non-glucocorticoid cohort identified as white. Full lists of pre- and post-match demographics and comorbidities are summarized in Table 1 and Table 2.

**Table 1:** Baseline Patient Characteristics Before Propensity Score Matching This table displays the baseline characteristics and demographics of the patients in the cohorts before propensity score matching. An alpha level of 0.05 was selected for statistical significance. SD, standard deviation; BMI, body mass index

Baseline Characteristic	Received Glucocorticoids	% of Cohort	Received No Glucocorticoids	% of Cohort	P-Value
Patients	67741		26938		
Age at Index	61.0 (13.10 SD)		58.6 (14.20 SD)		0.00
Male	30614	45.19%	13069	48.52%	0.00
Female	36752	54.25%	13823	51.31%	0.00
White	55257	81.57%	22117	82.10%	0.56
Black or African American	5383	7.95%	2058	7.64%	0.11
Asian	1138	1.68%	320	1.19%	0.00
American Indian or Alaska Native	202	0.30%	76	0.28%	0.68
Native Hawaiian or Other Pacific Islander	145	0.21%	15	0.06%	0.00
Unknown Race	11619	17.15%	5076	18.84%	0.00
Not Hispanic or Latino	3040	4.49%	1623	6.03%	0.00
Hispanic or Latino	53082	78.36%	20239	75.13%	0.00
Unknown Ethnicity	5616	8.29%	2352	8.73%	0.03
Essential (primary) Hypertension	34649	51.15%	10430	38.72%	0.00
Diabetes Mellitus	13419	19.81%	4626	17.17%	0.00
Overweight and Obesity	16745	24.72%	4007	14.88%	0.00
Nicotine Dependence	10756	15.88%	3253	12.08%	0.00
Osteopenia	7486	11.05%	1879	6.98%	0.00
Osteoporosis	5976	8.82%	1476	5.48%	0.00
Symptoms and Signs Concerning Food and Fluid Intake	5298	7.82%	1260	4.68%	0.00
Other Chronic Obstructive Pulmonary Disease	3471	5.12%	927	3.44%	0.00
Heart Failure	4302	6.35%	815	3.03%	0.00
Unspecified Kidney Failure	2784	4.11%	427	1.59%	0.00
BMI 40.0-44.9, Adult	2160	3.19%	320	1.19%	0.00
BMI 30.0-30.9, Adult	2121	3.13%	288	1.07%	0.00
BMI 31.0-31.9, Adult	2146	3.17%	320	1.19%	0.00
BMI 32.0-32.9, Adult	1978	2.92%	299	1.11%	0.00
BMI 33.0-33.9, Adult	2052	3.03%	284	1.05%	0.00
BMI 34.0-34.9, Adult	1929	2.85%	295	1.10%	0.00
BMI 35.0-35.9, Adult	539	0.80%	85	0.32%	0.00
BMI 50.0-59.9, Adult	352	0.52%	90	0.33%	0.00
BMI 70 or Greater, Adult	37	0.06%	10	0.04%	0.28
BMI 60.0-69.9, Adult	77	0.11%	11	0.04%	0.00

**Table 2:** Baseline Patient Characteristics After Propensity Score Matching This table displays the baseline characteristics and demographics of the patients in the cohorts after propensity score matching. An alpha level of 0.05 was selected for statistical significance. SD, standard deviation; BMI, body mass index

Baseline Characteristic	Received Glucocorticoids	% of Cohort	Received No Glucocorticoids	% of Cohort	P-Value
Patients	26903		26903		
Age at Index	58.7 (14.00 SD)		58.6 (14.10 SD)		0.56
Male	12861	47.81%	13046	48.49%	0.11
Female	13989	52.00%	13811	51.34%	0.12
White	22229	82.63%	22090	82.11%	0.12
Black or African American	1982	7.37%	2057	7.65%	0.22
Asian	334	1.24%	320	1.19%	0.58
American Indian or Alaska Native	51	0.19%	76	0.28%	0.03
Native Hawaiian or Other Pacific Islander	14	0.05%	15	0.06%	0.85
Unknown Race	4994	18.56%	5075	18.86%	0.37
Not Hispanic or Latino	1407	5.23%	1590	5.91%	0.00
Hispanic or Latino	20502	76.21%	20238	75.23%	0.01
Unknown Ethnicity	2293	8.52%	2345	8.72%	0.42
Essential (Primary) Hypertension	10324	38.38%	10430	38.77%	0.35
Diabetes Mellitus	4473	16.63%	4620	17.17%	0.09
Overweight and Obesity	4004	14.88%	4007	14.89%	0.97
Nicotine Dependence	3118	11.59%	3253	12.09%	0.07
Osteopenia	1791	6.66%	1879	6.98%	0.13
Osteoporosis	1394	5.18%	1476	5.49%	0.12
Symptoms and Signs Concerning Food and Fluid Intake	1171	4.35%	1260	4.68%	0.06
Other Chronic Obstructive Pulmonary Disease	790	2.94%	927	3.45%	0.00
Heart Failure	762	2.83%	815	3.03%	0.18
Unspecified Kidney Failure	465	1.73%	427	1.59%	0.2
BMI 40.0-44.9, Adult	308	1.15%	320	1.19%	0.63
BMI 30.0-30.9, Adult	305	1.13%	288	1.07%	0.48
BMI 31.0-31.9, Adult	297	1.10%	320	1.19%	0.35
BMI 32.0-32.9, Adult	286	1.06%	299	1.11%	0.59
BMI 33.0-33.9, Adult	283	1.05%	284	1.06%	0.97
BMI 34.0-34.9, Adult	273	1.02%	295	1.10%	0.35
BMI 35.0-35.9, Adult	89	0.33%	85	0.32%	0.76
BMI 50.0-59.9, Adult	68	0.25%	90	0.34%	0.08
BMI 70 or Greater, Adult	10	0.04%	10	0.04%	1.00
BMI 60.0-69.9, Adult	10	0.04%	11	0.04%	0.83

Outcomes

The data for the cohorts' outcomes, after propensity score matching, is displayed in Table 3. After propensity score matching, the glucocorticoid-exposed cohort had higher odds of reoperation, deep SSI, superficial SSI, and ED visits within 30 days of operation compared to the non-glucocorticoid-exposed cohort. The glucocorticoid-exposed cohort also had significantly lower odds of developing pneumonia, tracheostomy requirement, and renal insufficiency compared to the non-glucocorticoid-exposed cohort. There were no significant differences between the cohorts’ odds of death, DVT/PE, MI, UTI, AKI, sepsis, reintubation, and ventilator dependence.

**Table 3:** Postoperative Complications With Glucocorticoid Use This table displays the odds ratios of postoperative complications occurring within 30 days of the operation. An alpha level of 0.05 was selected for statistical significance. SSI, surgical site infection; AKI, acute kidney injury; PE, pulmonary embolism; DVT, deep vein thrombosis; UTI, urinary tract infection; ED, emergency department; MI, myocardial infarction; CI, confidence interval

Complication	Odds Ratio	95% Cl Lower	95% Cl Upper	P-Value
Death	0.948	0.744	1.208	0.665
Reoperation	1.352	1.111	1.645	0.003
SSI - Superficial	2.279	1.571	3.307	0.000
SSI - Deep	1.860	1.314	2.633	0.000
Pneumonia	0.685	0.591	0.793	0.000
Reintubation	0.779	0.565	1.072	0.124
Ventilator Dependence	0.797	0.559	1.136	0.208
Tracheostomy	0.391	0.255	0.601	0.000
AKI	1.021	0.900	1.159	0.747
Renal Insufficiency	0.804	0.651	0.993	0.042
PE/DVT	0.957	0.853	1.073	0.448
UTI	0.927	0.827	1.039	0.192
ED Visit	1.250	1.172	1.333	0.000
Sepsis	0.901	0.758	1.070	0.235
MI	0.897	0.708	1.136	0.366

## Discussion

This retrospective study of 67741 patients who underwent single-level lumbar fusions demonstrated increased odds of reoperation, deep and superficial SSI, and ED visits among patients who used glucocorticoids for at least a week in the year preceding surgery. However, these patients who used glucocorticoids also demonstrated decreased odds of developing pneumonia, renal insufficiency, and tracheostomy requirement.

Glucocorticoids are commonly used to manage various inflammatory, autoimmune, neoplastic, and other medical conditions. Their anti-inflammatory properties are well-described and predominantly exerted by blocking the release of inflammatory mediators such as interleukin (IL)-1-alpha and IL-1-beta, while inducing the release of anti-inflammatory mediators such as I-kappa-B-alpha, lipocortin-1, IL-10, and alpha-2-macroglobulin [20]. Furthermore, glucocorticoids promote the resolution of inflammatory vasodilation, vascular permeability, leukocyte emigration to inflamed sites, and immune cell differentiation [21-23]. Additionally, glucocorticoids dampen signal transduction downstream of cytokine pattern recognition Fcɛ receptors and promote resolution of inflammatory response through programming effects on macrophages [24]. However, due to their immunosuppressive effects, glucocorticoids may increase host susceptibility to viral, bacterial, fungal, and parasitic infections [25-28]. The immunosuppression and subsequent increased infection risk associated with glucocorticoid use are thought to result from impaired phagocytosis and suppression of humoral immunity (decreased function of B-lymphocytes) and cell-mediated immunity through inhibition of pro-inflammatory genes such as NF-kB and AP-1 through trans-repression [12,21,29-37]. Glucocorticoids regulate adaptive immunity by inhibiting lymphocyte activation and promoting lymphocyte apoptosis. Newer models have expanded to highlight the anti-inflammatory effects of glucocorticoids at both transcriptional and post-transcriptional levels through combinations of gene activation and inhibition [21,38].

These immunosuppressive effects likely explain the increased odds of deep and superficial SSI and reoperation in the cohort that was exposed to glucocorticoids. High doses of glucocorticoids have been found to have an inhibitory effect on the production of B- and T-cells, increasing the odds of developing SSI. Studies have also proposed that low endogenous concentrations of glucocorticoids sensitize the innate immune system, while high concentrations suppress it through attenuated intracellular signaling [24]. The increased odds of developing SSI relate directly to increased odds of reoperation, including wound revisions, washouts, and removal of spinal fusion implants. Furthermore, suppression of inflammatory cascades, affecting blood flow and leukocyte migration, impairs immunological mechanisms of acute wound healing and increases the risk of complications, including reoperation [39]. All these effects of glucocorticoids, increasing infection rates, hindering wound healing, and increasing need for reoperation, often initially present as ED visits. This aligns with the results of this study, suggesting that preoperative glucocorticoid use increases the odds of requiring ED visits.

The attenuation of the inflammatory cascades and immune cell migration by glucocorticoid use in the preoperative period likely explains the cohort's decreased odds of developing pneumonia, renal insufficiency, and tracheostomy requirement [20-24,40]. Glucocorticoids have been found to decrease renal damage and, therefore, the odds of developing renal insufficiency by limiting neutrophil degranulation and its damaging effects [41]. This aligns with the results of this study, suggesting that preoperative glucocorticoid use reduces the odds of developing renal insufficiency.

A review by Cutolo et al. demonstrated that the odds of developing infection depend primarily on the dose of glucocorticoids and tend to remain low in patients exposed to low peak-dose levels, even despite high cumulative doses [30]. As a result, in certain chronic immune or inflammatory conditions in which cortisol secretion is inadequate, glucocorticoid therapy can dramatically decrease the odds of developing infections, including pneumonia [30]. Results from a separate clinical trial suggest that a patient’s underlying disease state accounts for a portion of the presumed steroid-associated infectious complications [31]. A study by Abe et al. reported that even glucocorticoid usage at low dosages could increase the odds of developing infection, particularly in patients with systemic lupus erythematosus [42]. Additionally, although doses of long-term corticosteroids have decreased over the past several decades due to costly adverse events, dose reduction may not be a sufficient solution depending on a patient’s pathology [43].

The data from this study, demonstrating increased odds of reoperation, deep and superficial SSI, and ED visits, aligns with the existing literature suggesting a relationship between chronic steroid use and subsequent surgical complications noted in gastrointestinal surgery, neurosurgery, orthopedics, and the specific area of spine surgery [7-9,12,13,19,38,43-62]. Kantar et al. reported that patients using glucocorticoids for at least 30 days prior to surgery were at increased risk of postoperative venous thromboembolism; all-cause mortality; UTIs, sepsis; and wound, cardiac, and pulmonary complications [63]. From analysis of data on 5441 patients from the American College of Surgeons National Surgical Quality Improvement Program who underwent posterior cervical surgery, including posterior cervical decompression, fusion, and cervical laminoplasty, Sebastian et al. reported that long-term corticosteroid use was an independent risk factor for SSI [60]. Lieber et al. found similar associations but with preoperative steroid use greater than 10 days [63]. Ranson et al. found that chronic preoperative steroid use was associated with an increased risk of deep SSI, superficial SSI, wound dehiscence requiring reoperation, UTI, PE, non-home discharge, and ED visits or readmission following elective posterior lumbar fusion (PLF) [19]. Meanwhile, Cloney et al. demonstrated that preoperative corticosteroid therapy was associated with a moderately increased risk of infection-related complications (OR=1.65) within 30 days following PLF. Still, they found no associations with readmission or reoperation rates [61]. Additionally, Tihista et al. reported that long-term corticosteroid usage was associated with a significantly increased risk of acute postoperative complications, including UTIs, sepsis and septic shock, thromboembolic complications, and extended length of hospital stay, but not with deep or superficial SSIs [12]. Kebaish et al. reported significant increases in perioperative adverse outcomes for patients using glucocorticoids and undergoing elective posterior lumbar surgery, even with matching and controlling for potentially confounding variables [13]. White et al. reported that preoperative steroid therapy was associated with perioperative complications such as deep SSIs, pulmonary complications, requirement of blood transfusions, and extended length of stay following anterior lumbar fusion [62].

The results of this study demonstrated no significant differences in odds of mortality between cohorts. This result is consistent with those of a multicenter, double-blind, randomized controlled trial performed by Asehnoune et al., which demonstrated patients who received 0.2 mg/kg of dexamethasone preoperatively had no significant differences in rates of complications or mortality within 14 days after surgery [64]. Other studies have demonstrated increases in mortality associated with preoperative steroid use [62,63]. These conflicting results may be explained by the well-demonstrated dose-dependent effects of steroids and the varying pathologies indicating their use [24,30]. These results highlight the need for an interdisciplinary approach to optimize the medical management of patients requiring glucocorticoid therapy and planning to undergo surgery.

Our study had several limitations. The TriNetX database does not indicate the medical conditions or diagnoses indicating glucocorticoid use. The database also does not indicate the dosage, mode of administration, frequency, or specific type of glucocorticoids used. These characteristics may lead to potential confounding factors. Furthermore, as complications were only queried 30 days postoperation, our team could not evaluate the long-term outcomes associated with preoperative glucocorticoid usage.

## Conclusions

Patients with at least a week of exposure to glucocorticoids in the year prior to single-level spinal fusion surgery were found to have significantly increased odds of experiencing postoperative complications, including reoperation, deep and superficial SSIs, and ED visits. However, these same patients had significantly lower odds of developing pneumonia, renal insufficiency, and tracheostomy requirement. Our findings confirmed that preoperative glucocorticoid therapy usage decreases and increases the odds of developing various surgical complications and should warrant patient-specific counseling. The risks and benefits of glucocorticoids before surgery, specifically spinal fusion surgery, should be weighed for patients, considering their risk profiles. Further studies should be conducted to confirm the findings of our study.
